# Transcorneal Tube Erosion of an Ahmed Valve Implant in an Adult

**DOI:** 10.4103/0974-9233.71593

**Published:** 2010

**Authors:** Sami Al-Shahwan

**Affiliations:** Glaucoma Division, King Khaled Eye Specialist Hospital, P.O. Box 7191, Riyadh 11462, Saudi Arabia

**Keywords:** Ahmed Valve Implant, Glaucoma Drainage Implant, Intraocular Pressure

## Abstract

Ahmed valve implants are currently used to manage high-risk complicated adults and pediatric glaucoma when standard filtration surgery is unsuccessful. Despite its success, the Ahmed valve shunt has significant complications particularly in the anterior segment. We report an unusual case of transcorneal tube erosion of an Ahmed valve implant in an adult that resulted from long-standing tube-corneal touch. Periodic observation of tube position is recommended.

## INTRODUCTION

Glaucoma drainage implants (GDIs) have become an important method of controlling intraocular pressure (IOP) in patients with refractory glaucoma. Surgery with GDIs is associated with similar operative and postoperative complications that may occur after filtering surgery such as hypotony, hyphema, cataract, corneal decompensation, and failure to control IOP.^1^ In addition, several unique complications may develop with GDIs related to the presence of an implanted foreign body such as diplopia,^2^ conjunctival,^3^ and transcorneal tube erosion.^4^ We report an unusual case of transcorneal tube erosion following Ahmed valve implant.

## CASE REPORT

A 19-year-old male developed an uncontrolled IOP after retinal detachment repair in the left eye for which he underwent an Ahmed valve implant with an overlying pericardial patch graft.

The plate of the implant was placed in the superonasal quadrant and sutured to the posterior edge of the scleral buckle. Moderate scarring was noted during dissection. Postoperatively, the IOP was controlled and the visual acuity was 20/100. The tube appeared well positioned and away from the corneal endothelium.

**Figure 1:**
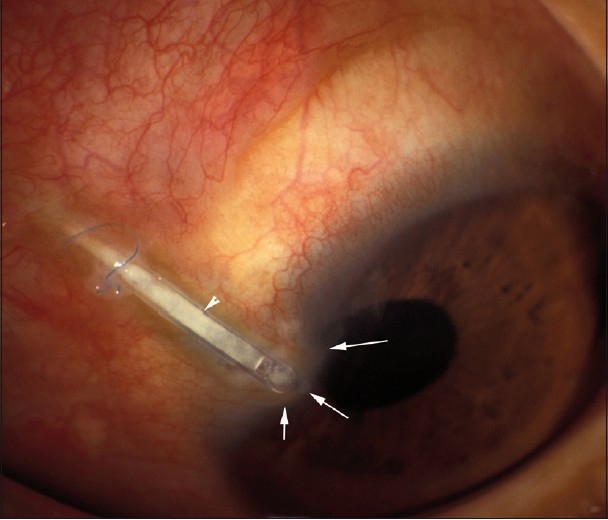
Photomicrograph demonstrating transconjunctival (arrowhead) and corneal erosion of the tube as evidenced by corneal thinning and full thickness opacity (arrows) surrounded by hyperemic conjunctiva. Note the broken prolene anchoring suture at the site of conjunctival erosion

Ten months later, the patient returned complaining of foreign body sensation, redness, tearing, and mild ocular tenderness for several days. On examination, visual acuity was 20/200 in the left eye and IOP was 29 mmHg. Slit lamp examination revealed an extruded tube with melting of the pericardial patch, conjunctival erosion, and sterile corneal infiltrate adjacent to the tube [Figure 1] that was Seidel negative with mucous secretion partially covering the area. The anterior chamber was deep with a mild anterior chamber reaction. The remaining examination was unremarkable. During the removal of the tube and plate, the anterior chamber remained deep without leakage at the limbus or the area of corneal opacity. The plate appeared to in normal position without migration.

**Figure 2:**
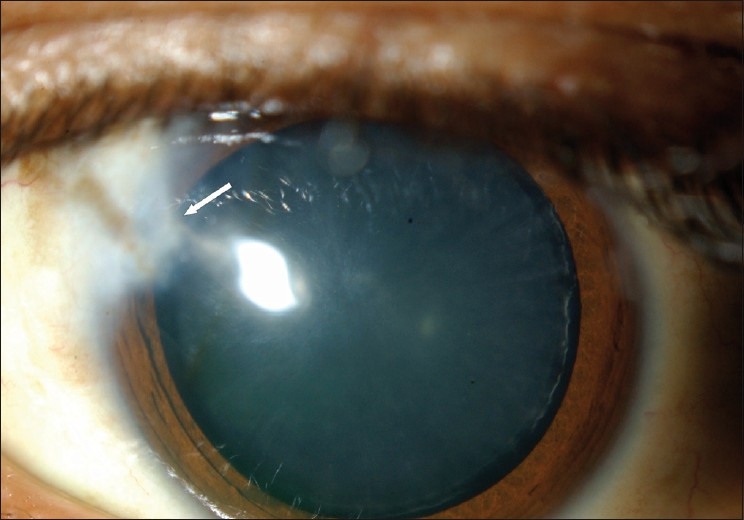
The anterior segment of the left eye following removal of the extruded tube. A triangular corneal opacity in the area of erosion is seen (arrow)

The patient underwent diode cyclophotocoagulation to control his IOP. At the last follow-up visit, the area of repair appeared well healed; the conjunctiva was intact with a persistent corneal scar [Figure 2].

## DISCUSSION

This case demonstrates a rare complication of the tube portion of the shunt in a 19-year-old male. While transcorneal erosion and/or migration in children has been described in two reports,^4^,^5^ this is not surprising because the rate of tube exposure in pediatric shunts (10–13%)^6^,^7^ is five times higher than the rate reported for glaucoma implants in adults (0–2%).^8^,^9^

Although the incidence of tube erosion has decreased after covering glaucoma drainage tubes by donor patch grafts,^3^ the causes of tube erosion, which have not been well defined, are probably due to inflammatory, immune-mediated melting of graft materials.^10^

Several factors may have predisposed the tube to erode in our patient. Extensive pre-existing surgical scarring, from previous scleral buckle surgery, might have caused progressive localized tenting of the plate and the tube causing tube-cornea touch during the 10 months when the patient was lost to follow-up. It is also possible that the superonasal location of the plate where space is limited may have caused excessive anterior tenting of the tube despite the tube being anchored with prolene along its length, leading to tube-cornea touch. Tube touch resulting in local corneal endothelial decompensation might have prompted a toxic reaction as suggested by the inflammatory reaction around the tube in our case. These events might have led to slow corneal erosion with concurrent scar formation posterior to the eroding tube, thereby preventing aqueous humor leakage. Positioning tubes as posteriorly as possible in the anterior chamber or in other locations such as inferonasal quadrant may reduce incidence of this complication. Periodic observation of tube position, especially in patients with previous surgery who had extensive scarring, is highly recommended.
